# Inflammatory Markers Used as Predictors of Subclinical Atherosclerosis in Patients with Diabetic Polyneuropathy

**DOI:** 10.3390/life13091861

**Published:** 2023-09-03

**Authors:** Adrian Vasile Mureșan, Alexandru Tomac, Diana Roxana Opriș, Bogdan Corneliu Bandici, Cătălin Mircea Coșarcă, Diana Carina Covalcic, Ioana Hălmaciu, Orsolya-Zsuzsa Akácsos-Szász, Flavia Rădulescu, Krisztina Lázár, Adina Stoian, Mariana Cornelia Tilinca

**Affiliations:** 1 Clinic of Vascular Surgery, Mures County Emergency Hospital, 540136 Targu Mures, Romania; adrian.muresan@umfst.ro (A.V.M.); catalin.cosarca@umfst.ro (C.M.C.);; 2 Department of Vascular Surgery, George Emil Palade University of Medicine, Pharmacy, Science and Technology of Targu Mures, 540139 Targu Mures, Romania; 3 Clinic of Plastic Surgery, Emergency Clinical Hospital Saint Spiridon, 700111 Iasi, Romania; alex.tomac@yahoo.com; 4 Emergency Institute for Cardiovascular Diseases and Transplantation (IUBCVT) of Targu Mures, 540136 Targu Mures, Romania; 5 Department of Anatomy, George Emil Palade University of Medicine, Pharmacy, Science and Technology of Targu Mures, 540139 Targu Mures, Romania; 6 Department of Radiology, Mures County Emergency Hospital, 540136 Targu Mures, Romania; ioana.halmaciu@umfst.ro; 7 Doctoral School of Medicine and Pharmacy, George Emil Palade University of Medicine, Pharmacy, Science and Technology of Targu Mures, 540142 Targu Mures, Romania; 8 Clinical Department of Endocrinology, Mures County Emergency Hospital, 540136 Targu Mures, Romania; flavia.maria1290@gmail.com (F.R.);; 9 Department of Scientific Research Methodology, George Emil Palade University of Medicine, Pharmacy, Science and Technology of Targu Mures, 540139 Targu Mures, Romania; 10 Department of Pathophysiology, George Emil Palade University of Medicine, Pharmacy, Science and Technology of Targu Mures, 540139 Targu Mures, Romania; adina.stoian@umfst.ro; 11 Department of Diabetes, Nutrition and Metabolic Diseases, Mures County Emergency Hospital, 540136 Targu Mures, Romania; mariana.tilinca@umfst.ro; 12 Department of Internal Medicine, George Emil Palade University of Medicine, Pharmacy, Science and Technology of Targu Mures, 540139 Targu Mures, Romania

**Keywords:** inflammatory markers, diabetic polyneuropathy, atherosclerosis, MLR, NLR, PLR, SII

## Abstract

Background: peripheral arterial disease (PAD) is identified late in diabetic patients because, in the majority of cases, it is associated with diabetic peripheral neuropathy, resulting in little or no symptoms, or symptoms that are completely neglected. Methods: In this study were enrolled all patients over 18 years of age, with diabetes mellitus type II for more than a year with poor glycemic control, diagnosed with diabetic polyneuropathy admitted to the Diabetology Department, Emergency County Hospital of Targu Mures, Romania between January 2020 and March 2023. We divided the patients into two groups, based on the presence or absence of subclinical atherosclerosis in the lower limb, named “SA” and “non-SA”. Results: Patients in the SA group were older (*p* = 0.01) and had a higher incidence of IHD (*p* = 0.03), history of MI (*p* = 0.02), and diabetic nephropathy (*p* = 0.01). Moreover, patients with subclinical atherosclerosis had a higher BMI (*p* < 0.0001) and a longer duration of diabetes (*p* < 0.0001). Among all patients, the systemic inflammatory markers, MLR (r = 0.331, *p* < 0.001), NLR (r = 0.517, *p* < 0.001), PLR (r = 0.296, *p* < 0.001), SII (r = 0.413, *p* < 0.001), as well as BMI (r = 0.241, *p* < 0.001) and HbA1C (r = 0.489, *p* < 0.001), demonstrated a strong positive correlation with the diabetes duration. The multivariate logistic regression analysis showed that older patients (OR: 2.58, *p* < 0.001), the male gender (OR: 2.30, *p* = 0.006), a higher baseline levels of BMI (OR: 7.71, *p* < 0.001), and the duration of diabetes (OR: 8.65, *p* < 0.001) are predictors of subclinical atherosclerosis in DN patients. Additionally, the high baseline levels of all systemic inflammatory markers (for all: *p* < 0.001) and poor diabetes management (OR: 10.4, *p* < 0.001 for HbA1C; OR: 10.78, *p* < 0.001 for admission glucose) are independent predictors of SA. Conclusions: the inflammatory markers, NLR, MLR, PLR, and SII, being cheap and easy to collect in routine medical practice from the standard blood tests, could be an important step in predicting vascular outcomes in diabetic patients and the disease’s progression, playing a key role in follow-up visits in type-2 diabetic patients and PAD patients.

## 1. Introduction

A continuously aging population and their continuous variations in alimentation patterns and day-to-day life culminate in an increase in the frequency, disability, and mortality rates of diabetes, the primary causes of which are defective insulin secretion or insufficient feedback to insulin, having negative consequences on the world’s public health and demanding tremendous efforts in changing and adapting the health policies to this progressively demanding situation. The number of people suffering from diabetes around the world has increased by almost 300% in the last 30 years [1]. Furthermore, in 2019, almost 9.3% of the adult population was suffering from diabetes, which equates to 463 million people’s lives being harmed daily by this “silent killer” [2].

With an estimated global population of over 200 million people [3], peripheral arterial disease (PAD) is characterized by the presence of atherosclerotic plaques in the arteries of the lower limbs. Diabetes mellitus (DM) is a key risk factor for the development of PAD and an independent predictor of low-extremity amputations and death in PAD patients [4,5,6]. Following the Framingham study, it is known that DM is a significant risk factor for PAD [7], and the damaged region is more widespread and clinically much more severe in DM patients [4,5,6,7]. Furthermore, PAD patients with diabetes exhibit infrainguinal and crural Trans-Atlantic Inter-Society Consensus (TASC) II C/D lesions, which have a highly unsatisfactory evolution with poor long-term results [7].

PAD is diagnosed late in diabetic patients because, in the majority of cases, it is accompanied by diabetic peripheral neuropathy, resulting in few symptoms or being completely neglected. Because PAD is asymptomatic in its early stages and results in reduced pain perception in diabetic patients due to peripheral neuropathy that affects sensory feedback [8], it is predicted that only 20–30% of those with DM develop PAD [9].

When it comes to the mechanism of PAD in diabetic patients, it associates the pathological states encountered in DM (hyperglycemia, insulin resistance) with those encountered in PAD (vascular inflammation, endothelial cell dysfunction, anomalies in blood cells, including smooth muscle cells and platelets), all of which contribute to the increased plaque burden and its instability, as well as the disease’s and treatment’s increased complexity [10,11].

A constant level of elevated blood glucose and insulin resistance can cause significant vascular deterioration, resulting in neuropathy, nephropathy, retinopathy, and cardiovascular diseases, including a course of peripheral arterial disease; they all contribute to both the mortality and morbidity of diabetes mellitus type 1 and 2, [12], a diabetic patient having a risk up to three times higher of premature death with their life expectancy being shorter by 8 years [13]. Diabetic peripheral neuropathy is the most prevalent chronic neurological consequence [14], affecting half of all patients, with distal symmetric polyneuropathy being the most common manifestation.

In recent years, there has been an increase in scientific research that emphasizes the standard blood testing that can provide data about certain diseases [15,16]. There is evidence that neutrophile-to-lymphocyte ratio (NLR), monocyte-to-lymphocyte ratio (MLR), and platelet-to-lymphocyte ratio (PLR) predict the progression of complications in diabetic patients [17,18,19,20,21,22,23], as well as their function in predicting cardiovascular events [13,14,15,16]. The purpose of this study is to evaluate the association between inflammation and, by using WBC-derived inflammatory biomarkers, to try to predict subclinical atherosclerosis in patients with peripheral diabetic neuropathy.

## 2. Materials and Methods

### 2.1. Study Design

In this observational, retrospective study were enrolled all patients over 18 years of age, with diabetes mellitus type II for over one year, diagnosed with diabetic polyneuropathy admitted to the Diabetology Department, Emergency County Hospital of Targu Mures, Romania between January 2020 and March 2023. Patients with oncological disease, hematological disease, history of major surgery in the last 3 months, and symptomatic atherosclerosis of the lower limb were excluded. Based on the diagnosis of subclinical atherosclerosis in the lower limb, we divided the patients into two groups with and without subclinical atherosclerosis named “SA” and “non-SA”.

### 2.2. Data Collection

We extracted, from the hospital’s electronic database, the demographic data, comorbidities, and laboratory analyses on the day of admission: hemoglobin level, hematocrit level, number of neutrophils, monocytes, platelets, lymphocytes, glucose level, total cholesterol level, triglyceride level, blood urea nitrogen (BUN), creatinine, and glomerular filtration rate (GFR).

### 2.3. Study Outcomes

The primary outcomes were the risk of subclinical atherosclerosis at the level of the lower limb in patients with diabetic polyneuropathy with poor glycemic control. Subclinical atherosclerosis at the level of the lower limb was quantified as any 50–70% stenosis at the level of the superficial femoral artery or the popliteal artery found in computed tomography angiography exams.

### 2.4. Statistical Analysis

SPSS for Mac OS version 28.0.1.0 was used for statistical analysis (SPSS, Inc., Chicago, IL, USA). Chi-square tests were used to assess the associations of the ratios with category factors, while Student’s t- or Mann–Whitney tests were used to assess differences in continuous variables. The ROC curve analysis was used to determine the appropriate BMI, admission glucose level, HbA1C, NLR, MLR, PLR, and SII cut-off values based on the Youden index (Youden Index = sensitivity + specificity − 1, ranging from 0 to 1). To identify independent predictors of SA in patients with DN, a multivariate logistic regression analysis using variables with *p* < 0.1 was undertaken.

## 3. Results

During the research period, 198 patients met the criteria and were enrolled in this study, with an average age of 64.36 ± 10.18, of which 105 patients were female (53.03%), and 71 patients (36.36%) had subclinical atherosclerosis in the lower limb. Moreover, the patients in the SA group were older (*p* = 0.01). Regarding the comorbidities and risk factors, as we see in Table 1, there was a greater incidence of IHD (*p* = 0.03), history of MI (*p* = 0.02), as well as diabetic nephropathy (*p* = 0.01) in the second group of patients. Moreover, patients with subclinical atherosclerosis had a higher BMI (*p* < 0.0001) and a longer duration of diabetes (*p* < 0.0001).

In addition, statistically significant variations in laboratory analyses were found between the two groups. As a result, we recorded higher values for HbA1C (*p* < 0.0001), admission glucose (*p* < 0.0001), cholesterol (*p* < 0.0001), creatinine (*p* = 0.02), neutrophil (*p* = 0.02), and all the WBC-derived inflammatory markers (all *p* < 0.0001). On the other hand, we recorded a low level of monocytes (*p* = 0.03), lymphocytes (*p* < 0.0001), ALT (*p* = 0.04), and AST (*p* = 0.007).

**Figure 1:**
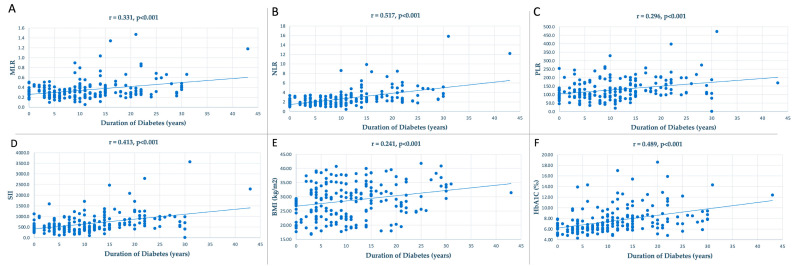
Plot representation of the dispersion of data of the correlation between duration of diabetes (years) and (**A**) MLR (r = 0.331, *p* < 0.001), (**B**) NLR (r = 0.517, *p* < 0.001), (**C**) PLR (r = 0.296, *p* < 0.001), (**D**) SII (r = 0.413, *p* < 0.001), (**E**) BMI (r = 0.241, *p* < 0.001), and (**F**) HbA1C (r = 0.489, *p* < 0.001).

Among all patients, systemic inflammatory markers, MLR (r = 0.331, *p* < 0.001), NLR (r = 0.517, *p* < 0.001), PLR (r = 0.296, *p* < 0.001), SII (r = 0.413, *p* < 0.001), as well as BMI (r = 0.241, *p* < 0.001) and HbA1C (r = 0.489, *p* < 0.001), exhibited a strong positive correlation with the duration of diabetes, as seen in Figure 1.

**Figure 2:**
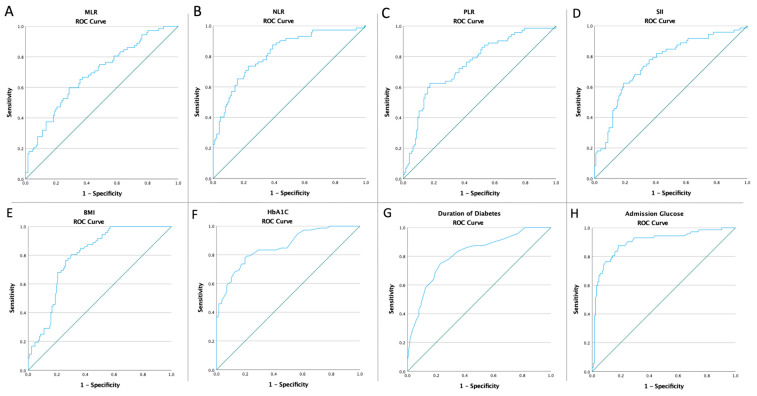
The ROC curve graphics regarding the prediction of SA in PN patients for (**A**) MLR (AUC:0.689, *p* < 0.0001), (**B**) NLR (AUC:0.820, *p* < 0.0001), (**C**) PLR (AUC:0.751, *p* < 0.0001), (**D**) SII (AUC:0.759, *p* < 0.0001), (**E**) BMI (AUC:0.793, *p* < 0.0001), (**F**) HbA1C (AUC:0.857, *p* < 0.0001), (**G**) duration of diabetes (AUC:0.812 *p* < 0.0001), and (**H**) admission glucose (AUC:0.899, *p* < 0.0001).

When analyzing the ROC curve, as seen in Figure 2, all inflammatory markers (for all *p* < 0.0001), BMI (*p* < 0.0001), HbA1C (*p* < 0.0001), duration of diabetes (*p* < 0.0001), and glucose admission (*p* < 0.0001) were associated with subclinical atherosclerosis. Moreover, Table 2 presents the optimal cut-off value of the above-mentioned markers, as well as the characteristics.

The multivariate logistic regression analysis showed that older patients (OR: 2.58, *p* < 0.001), the male gender (OR: 2.30, *p* = 0.006), a higher baseline value of BMI (OR: 7.71, *p* < 0.001), and the duration of diabetes (OR: 8.65, *p* < 0.001) are predictors of subclinical atherosclerosis in DN patients (Table 3). Additionally, the high baseline level of all systemic inflammatory markers (for all *p* < 0.001) and poor controlling status of diabetes (OR: 10.4, *p* < 0.001 for HbA1C; OR: 10.78, *p* < 0.001 for admission glucose) are independent predictors of SA.

## 4. Discussion

The main result of this study is that the total number of neutrophils, monocytes, lymphocytes, and platelets can predict AS in diabetic patients with PN. Also, our study found that older patients (*p* < 0.001), the male gender (*p* = 0.006), the presence of cardiac comorbidities (for all: *p* < 0.05), diabetes duration (*p* < 0.001), high BMI values (*p* < 0.001), and poor diabetes control (*p* < 0.001) are associated with AS in patients with PN.

The tissue stress generated by persistent hyperglycemia is a major cause of microvascular disease. Major clinical studies, such as the UK Prospective Diabetes Study (UKPDS) and the Diabetes Control of Complications Trial (DCCT), have demonstrated a clear link between microvascular disease and glucose control [24,25]. Microvascular disease manifests itself most prominently in tissues where glucose uptake is insulin-independent, such as the retina, kidney, and vascular endothelium, where the glucose level is nearly the same as in the blood. The disease’s progression is the result of a combination of oxidative stress induced by superoxide overproduction, direct glucose-mediated endothelium degradation, and the production of sorbitol and advanced glycation end-products caused by continuous hyperglycemia [26].

Inflammation raises the risk of atherosclerosis and thrombosis. CRP (C-reactive protein) has multiple roles in both the evolution of PAD and the abnormal glucose regulation [27]: it increases the production of procoagulant tissue factor, chemotactic substances, and leukocyte adhesion molecules; it affects the vascularization by prohibiting the endothelial nitric oxide synthase which normally produces nitric oxide (NO) by switching to a different pathway which is phosphoinositol-3-kinase dependent [8,10]; it alters the fibrinolysis by blocking the formation of PAI-1 (plasminogen activator inhibitor). These changes increase the probability of diabetic vascular walls developing atherosclerosis [28].

DM, which is accompanied by high levels of proinflammatory cytokines (TNF, IL-6) [29,30], promotes the attachment of leukocytes and platelets to the endothelial wall through the nuclear factor (NF) and endothelial cell adhesion molecules, resulting in some ideal conditions for thrombus formation [10,28,31].

Diabetic retinopathy (DR), which is classified into two stages, non-proliferative diabetic retinopathy (NPDR) and proliferative diabetic retinopathy (PDR), is a significant complication of diabetes that is the leading cause of vision loss in working-age populations.

Inflammation has a critical role in the evolution of diabetic retinopathy. Recurrent low-grade inflammation has been observed in diabetic patients with DR [32], with leukostasis being acknowledged to play an important role in the early stages of DR but also being related to endothelial damage. Schroder et al. were the first to report granulocyte and monocyte obstruction of the retinal microvasculature [33]. In diabetic patients, there was an increase in leukocyte adhesion and the expression of leukocyte b2-integrins CD11a, CD11b, and CD18 [34], but also, high levels of endothelial cell adhesion molecules such as intercellular adhesion molecule-1(ICAM-1), vascular cell adhesion molecule (VCAM-1), and selectins (E-selectin) were seen in diabetic patients [35,36], the last two being also correlated with the severity of DR [37]. According to studies, chemokines that influence the attraction and activation of leukocytes, such as monocyte chemotactic protein-1 (MCP-1), macrophage inflammatory protein-1alpha (MIP-1alfa), and MIP-1beta, are elevated in diabetes [38]. Moreover, there were findings about the correlation between the high levels of inflammatory cytokines (tumor necrosis factor alpha—TNF-alfa, interleukin 6—IL-6, IL-8, IL-1beta) and the severity of the DR [39,40]. The pathophysiology of diabetic retinopathy is very complicated, involving hyperglycemia, through several metabolic pathways, retinal microvasculopathy, and retinal degeneration, with enhanced mitochondrial fragmentation and cell apoptosis, as well as inflammation.

Diabetic nephropathy (DN), a very well-known consequence of long-term diabetes linked to its rising morbidity and mortality, is induced by the glomerulus’ chronic suffering caused by hypertension and hyperglycemia and is typically characterized by the thickening of the basement membrane, atrophy, and arteriosclerosis, resulting in the loss of renal function over time [41]. With the rise of people being diagnosed with diabetes, DN’s prevalence is also expected to grow [42,43].

DN was not first thought to be an inflammatory condition, but Tavafi [44] demonstrated the role of inflammation in its evolution. With more evidence in the recent years regarding the role of IL-1, IL-18, and IL-6 in the progression of DN [45,46,47], it has been reported that inflammation is an important part of initiating and continuing the nephropathy’s evolution, with monocytes, macrophages, and leukocytes being part of its pathogenesis [47].

Inflammation could be possibly activated by a biochemical, metabolic, or hemodynamic process present in DN [48], with the IL-18, IL-6, TNF-alfa, and TNF-beta1 being at high levels in the circulating blood supply [30]; their role in DN’s evolution has already been proved, with other studies reporting that their levels rise with the aggravation of the disease [49]. Meanwhile, studies show that the slowing down of the inflammatory process had a protective effect on the disease’s capacity to progress [50], reiterating the role of inflammation in nephropathy’s evolution. Aside from the proinflammatory and fibrogenic cytokines that harm the renal structure, there are also high levels of chemoattractant cytokines and adhesion molecules, all of this being important because they attract circulating leukocytes and facilitate their migration into the kidney tissue. With the last mentioned being also capable of producing cytokines and other inflammatory key substances, it contributes to the continuously developing inflammatory processes in the renal structures [44].

The most common type of neuropathy encountered in daily medical practice is distal symmetric sensorimotor polyneuropathy (DPN), which is asymptomatic in up to 50% of patients and affects both small and big fibers. It can consist of a wide range of clinical disorders, such as cranial nerve palsies, autonomic dysfunction, and mononeuropathies.

Clinical studies have recently revealed that long-term low-intensity inflammation plays an undeniably key role in DPN. Some studies on DPN patients without and with pain showed that the latter group had a higher level of inflammation as indicated by inflammatory markers and cytokines [51]. In another study performed by Magrinelli et al. [52], it was found that high levels of IL-6 and IL-10 (recognized as anti-inflammatory cytokines) were found in some DPN patients, corresponding to some anomalies in large nerve fibers. At the same time, it appears that there is a certain association between high levels of IL-6, IL-1, transforming growth factor-beta, and tumor necrosis factor and the evolution of nerve degeneration in DPN [53].

Nuclear factor-2 erythroid-related factor 2 which controls the management of detoxifying and antioxidant enzymes was found to be low in DPN patients, while the nuclear factor-kappa light chain enhancer of B cells, implicated in proinflammatory cytokines was reported as high; this situation could result in progressive inflammation, increased oxidative stress, poor blood-nerve supply, nerve harm and apoptosis, and pain hypersensitivity [54].

After a study in the population-based cross-sectional Cooperative Health Research in the region of Augsburg F4, it was brought to our attention that the levels of some cytokines covering IL-1 receptor antagonist (IL-1RA) were affirmatively associated with DPN [54].

Herder et al. [55] reported that systemic inflammation was able to predict the onset and progression of DPN over 6.5 years, that increased levels of IL-6, soluble intracellular adhesion molecule-1 (ICAM-1), plasma high-sensitivity C-reactive protein, TNF-alfa and IL-1RA, and low levels of adiponectin were associated with DPN, while elevated IL-1RA and ICAM-1 are connected to its evolution. Herder et al. also reported that vascular cell adhesion molecule-1, chemokines, and E-selectin are at high levels when it comes to DPN and its progression, and they advocated using IL-1RA and ICAM-1 as biomarkers to predict the DPN’s evolution in diabetic patients.

Between the two groups, we had a greater significant statistic mean age difference (66.80 ± 10.13), this being reported also by Eschol J et al. [55] and Lekshmi [56], who found that age is a predictor for PAD; therefore, PAD prevalence was positively correlated with age. Senior patients are at a higher risk of vascular illnesses because they do not pay so much attention to their health. Many researchers have focused on the underdiagnosis of PAD in diabetic patients [57].

Meanwhile, the PAD group had more patients with a history of myocardial infarction and ischemic heart disease (15.57% and 63.88% vs. 5.55% and 48.41%, *p* = 0.02 and *p* = 0.03), with PAD patients having a higher rate of comorbidities. A strong relationship between the previous history of cardiovascular disease and PAD was reported by Merino et al. [58] and by Velescu et al. [59], where PAD was related to an increased risk of cardiovascular death, major macrovascular events, and myocardial infarction.

In the scientific medical literature, significant attention has been given to hematological indices such as NLR (neutrophil-to-lymphocyte ratio), MLR (monocyte-to-lymphocyte ratio), and PLR (platelet-to-lymphocyte ratio), along with derived indices like SII (systemic immune-inflammation index), SIRI (systemic immune-inflammation response index), and AISI (all-inflammatory index). An analysis of recently published articles reveals that among these inflammatory markers, NLR, MLR, PLR, and SII have emerged as valuable predictors of vascular disease. Numerous works have highlighted their predictive potential in this context [60,61,62,63,64,65,66,67,68,69,70,71,72,73].

Recently, Wachsmann et al. [74,75] emphasized the importance of diabetes control and HbA1C in the favorable evolution one year after endovascular treatment in patients diagnosed with critical limb ischemia; much more recently in 2022, they published a systematic review in which they demonstrated the role of leukotrienes in cardiovascular atherosclerotic pathology.

The main strength of this study is the large number of systemic inflammatory markers analyzed in the detection of subclinical atherosclerosis in diabetic patients with diabetic polyneuropathy. In contrast, this study has certain limitations. Because this was a monocentric retrospective study, we urge that multicentric investigations with the tracking of inflammatory markers in dynamics be conducted in the future to improve the accuracy of their prognostic potential. Also, another limitation is the monitoring of the presence of subclinical atherosclerosis during hospitalization; in the future, we propose to monitor the patients in the long term and to quantify, with the help of arterial Doppler ultrasound and through regular checks every six months, the degree and progression of the atherosclerotic damage.

## 5. Conclusions

Given the data from our study and all the other scientific research that has examined this topic, we believe that these inflammatory markers, NLR, MLR, PLR, and SII, which are cheap and easy to collect in everyday medical practice from the routine blood test, could be an important step in predicting vascular outcomes in diabetic patients and the disease’s evolution, playing a key role in follow-up visits in type-2 diabetic patients and PAD patients.

## Figures and Tables

**Table 1:** The variables analyzed in this study presented for all patients and compared according to the presence of AS.

Variables	All Patients *n* = 198	No-SA *n* = 126	SA *n* = 72	*p*-Value
Age mean ± SD (min–max)	64.36 ± 10.18 (35–87)	62.96 ± 9.99 (35–86)	66.80 ± 10.13 (42–87)	0.01
Male/Female gender no. (%)	93 (46.97%) 105 (53.03%)	53 (42.06%) 73 (57.94%)	40 (55.56%) 32 (44.44%)	0.06
Comorbidities and Risk factors, no. (%)				
Arterial Hypertension	183 (92.42%)	116 (92.06%)	67 (93.05%)	0.79
Ischemic Heart Disease	107 (54.04%)	61 (48.41%)	46 (63.88%)	0.03
Chronic Venous Insufficiency	89 (44.94%)	61 (48.41%)	28 (38.88%)	0.19
Malignancy	28 (14.14%)	20 (15.87%)	8 (11.11%)	0.35
Active Smoking	81 (40.9%)	25 (34.72%)	56 (44.44%)	0.18
History of Stroke	11 (5.55%)	7 (5.55%)	4 (5.55%)	NS
History of Myocardial Infraction	18 (9.09%)	7 (5.55%)	11 (15.27%)	0.02
End Stage Kidney Disease	28 (14.14%)	15 (11.9%)	13 (18.05%)	0.23
Diabetic Retinopathy	54 (27.27%)	30 (23.80%)	24 (33.33%)	0.14
Diabetic Nephropathy	40 (20.2%)	19 (15.07%)	21 (29.16%)	0.01
Anthropometric Characteristics, median [Q1–Q3]				
BMI (kg/m^2^)	29.32 [23.92–33.68]	25.81 [21.16–30.45]	32 [30.1–35.36]	<0.0001
Abdominal circumferential (cm)	110 [100–120]	109 [100–118.25]	112 [101–121]	0.052
Duration of Diabetes (years)	10 [5–15]	7 [4–11]	15 [11.75–22]	<0.0001
Laboratory Findings, median [Q1–Q3]				
HbA1C (%)	6.83 [6–8.3]	6.2 [5.8–7]	8.55 [7.3–11.2]	<0.0001
Admission Glucose (mg/dL)	141 [114–199.75]	122 [101.25–143]	218 [174–271.25]	<0.0001
Cholesterol (mg/dL)	167.3 [132–203.47]	154.15 [122.1–190.85]	189.4 [157.3–209.32]	<0.0001
Triglyceride (mg/dL)	162.1 [118.32–241.72]	152 [116.15–237.9]	177.6 [120.55–248.9]	0.12
AST (IU/L)	21 [15.9–30.8]	22.45 [16.15–31.97]	19.2 [15.7–27.17]	0.04
ALT (IU/L)	21.95 [15.87–35.52]	23.15 [18–41]	19.7 [14.2–30.92]	0.007
GGT (IU/L)	34 [23–72]	37 [21–75]	31.5 [23–62]	0.26
BUN (mg/dL)	41.55 [32.42–56.67]	39.45 [30.45–56.35]	43 [35.57–56.77]	0.09
Creatinine (mg/dL)	0.95 [0.77–1.29]	0.91 [0.73–1.23]	1.02 [0.84–1.38]	0.02
Hemoglobin (g/dL)	13.5 [12.3–14.6]	13.3 [12.3–14.47]	13.5 [12.47–14.87]	0.18
Hematocrit %	40.65 [37.22–43.65]	40.55 [37.1–43.5]	40.7 [37.27–43.72]	0.35
WBC	8.06 [6.63–9.78]	8.18 [6.64–9.67]	7.69 [6.32–9.84]	0.21
Neutrophil	4.8 [3.83–6.12]	4.66 [3.82–5.79]	5.22 [3.92–7.01]	0.02
Monocyte	0.61 [0.51–0.76]	0.63 [0.53–0.77]	0.59 [0.48–0.71]	0.03
Lymphocyte	2.04 [1.53–2.65]	2.34 [1.93–2.97]	1.67 [1.31–1.98]	<0.0001
PLT	237 [200.25–290.75]	246.5 [205.25–290.75]	231.5 [198.5–201]	0.20
NLR	2.24 [1.77–3.15]	1.95 [1.60–2.50]	3.16 [2.41–4.66]	<0.0001
MLR	0.30 [0.22–0.40]	0.27 [0.21–0.35]	0.35 [0.27–0.46]	<0.0001
PLR	116.52 [91.2–162.7]	103.26 [84.4–128.89]	157.64 [113.28–194.59]	<0.0001
SII	575.5 [399.9–838.7]	468.6 [363.2–631.1]	802.9 [577.1–1004.9]	<0.0001

**Table 2:** The optimal cut-off value and the characteristics of all the markers analyzed with the ROC curve.

Variables	Cut-Off	AUC	Std. Error	95% CI	Sensitivity	Specificity	*p*-Value
Subclinical Atherosclerosis of Lower Limb							
MLR	0.33	0.689	0.039	0.613–0.765	59.7%	71.4%	<0.0001
NLR	2.53	0.820	0.031	0.759–0.881	73.6%	77%	<0.0001
PLR	137.21	0.751	0.036	0.680–0.821	62.5%	82.5%	<0.0001
SII	615.91	0.759	0.036	0.688–0.829	68.1%	73.8%	<0.0001
BMI	30.87	0.793	0.031	0.732–0.854	68.1%	79.4%	<0.0001
HbA1C	7.45	0.857	0.028	0.802–0.911	73.6%	82.5%	<0.0001
Duration of Diabetes	9.5	0.812	0.032	0.749–0.875	83.3%	65.1%	<0.0001
Admission Glucose level	161.5	0.899	0.025	0.850–0.948	80.6%	85.7%	<0.0001

**Table 3:** Predictors of subclinical atherosclerosis in DN patients.

	Subclinical Atherosclerosis		
	OR	95% CI	*p*-Value
Demographic Characteristics			
Age	2.58	1.79–5.93	<0.001
Male	2.30	1.26–4.19	0.006
Comorbidities and Risk factors			
Ischemic Heart Disease	1.57	0.85–2.18	0.11
History of Myocardial Infraction	1.26	0.76–2.87	0.20
Diabetic Nephropathy	1.95	0.93–4.55	0.052
Tobacco	1.33	0.70–3.46	0.18
Anthropometric Characteristics			
BMI (kg/m^2^)	7.71	3.57–14.90	<0.001
Abdominal circumferential (cm)	1.86	0.84–3.78	0.06
Duration of Diabetes (years)	8.65	4.35–16.78	<0.001
Inflammatory Markers			
NLR	7.46	3.38–13.58	<0.001
MLR	4.63	1.99–9.12	<0.001
PLR	5.89	2.62–11.88	<0.001
SII	6.09	2.68–12.16	<0.001
Diabetes Controlling Status			
HbA1C	10.4	8.27–28.74	<0.001
Admission Glucose	10.78	8.69–32.45	<0.001

## Data Availability

Data are available based on the request from corresponding authors.
